# Regulation, function and evolutionary structure of *St18*, encoding a Zinc-Finger Transcription Factor, in the oligodendrocyte lineage

**DOI:** 10.21203/rs.3.rs-11211282/v1

**Published:** 2026-10-29

**Authors:** Daniel Yamamoto, Adrien M. Vaquié, Tim Aberle, Michael Perry, Davod R. Shah, Diana Gold Diaz, Guy Lam, Zhaoyang Xu, Gregory D. Jordan, Gemma C. Girdler, Hannah M. Wüst, Laura Morcom, Aimee S. Wilson, Khalil S. Rawji, Penelope Dimas, Guoqiang Gu, Sarah A. Teichmann, Robin J.M. Franklin, Ragnhildur T. Karadottir, Michael Wegner, David H. Rowitch

**Affiliations:** 1 Cambridge Stem Cell Institute, Jeffrey Cheah Biomedical Centre, Cambridge Biomedical Campus, University of Cambridge, Cambridge, United Kingdom; 2 Department of Paediatrics, University of Cambridge, Cambridge, United Kingdom; 3 Institut für Biochemie, Friedrich-Alexander-Universität Erlangen-Nürnberg, Erlangen, Germany; 4 Department of Medicine, University of Cambridge, Cambridge, United Kingdom; 5 Departments of Pediatrics and Neurosurgery, Cedars-Sinai Guerin Children’s, Los Angeles, CA, USA; 6 Board of Governors Regenerative Medicine Institute, Cedars-Sinai Health Sciences University, Los Angeles, CA, USA; 7 Altos Labs, Cambridge Institute of Science, Cambridge, United Kingdom; 8 Department of Cell and Developmental Biology, Vanderbilt University School of Medicine, Nashville, TN, USA; 9 CIFAR Macmillan Multi-scale Human Programme, CIFAR, Toronto, Canada; 10 Department of Physiology, Faculty of Medicine, University of Iceland, Reykjavik, Iceland

**Keywords:** Oligodendrocytes, oligodendrocyte progenitor cells, transcription factors, RNA/ATAC-sequencing, evolution, myelination, St18, Myt1, Myt3, NFOL

## Abstract

Myelinating oligodendrocytes are first observed during evolution in vertebrates above jawless fishes. Oligodendrocyte progenitor cells (OPCs) undergo extensive transcriptional and morphological remodelling to initiate differentiation and myelination. We report that the zinc-finger transcription factor *St18* (aka *Myt3*) is upregulated during the OPC-to-newly formed oligodendrocyte (NFOL) transition. Single-nucleus RNA-sequencing (snRNA-seq) performed after lysolecithin-induced demyelination of the mouse spinal cord revealed that *St18* was acutely upregulated in remyelinating OPCs. In primary rat OPC cultures, *St18* overexpression promoted differentiation, whereas OPC conditional *St18* knockout delayed developmental myelination in the mouse brain. Chromatin accessibility profiling identified candidate *cis*-regulatory elements for oligodendroglial *St18* expression activated by core oligodendrocyte transcription factors, including SOX10, KLF9/13, and MYRF, and we confirmed that *St18* expression in OPCs is *Sox10*-dependent *in vivo*. Phylogenetic analysis suggests that *St18* arose via duplication of *Myt1* in early jawed vertebrates, coinciding with the emergence of myelinating oligodendrocytes. These findings define *St18* as a transcriptional marker of NFOLs and OLs and provide insight into the evolutionary regulation of central nervous system myelination.

## Introduction

Myelin, a multilamellar membrane produced by oligodendrocytes in the central nervous system (CNS), is a defining innovation of jawed vertebrates that enabled packing a larger density of neuronal axons and hence brain complexity. By insulating axons, myelin facilitates rapid and energy-efficient saltatory conduction, while also providing essential trophic and metabolic support required for long-term axonal integrity^1^. Beyond these classical functions, myelin is increasingly recognised as a dynamic structure that contributes to learning and memory^2^. Consequently, disruption of myelin has broad implications for CNS diseases, as evidenced by hypomyelinating leukodystrophies and demyelinating disorders such as multiple sclerosis (MS)^3^.

In the adult CNS, remyelination is mediated by oligodendrocyte progenitor cells (OPCs), a widely distributed and self-renewing population that responds to injury through proliferation, migration and differentiation^3^. This process generates newly formed oligodendrocytes (NFOLs), which subsequently mature into myelinating cells. However, remyelination frequently fails, particularly at the transition from OPCs to NFOLs, which represents a major bottleneck in regeneration^4,5^. NFOLs are especially vulnerable to extrinsic stressors, including inflammation and hypoxia, and exhibit impaired differentiation in both aged animals and MS lesions^6–8^. This stage therefore represents a critical regulatory checkpoint at which myelination can be either successfully completed or aborted.

Oligodendrocyte lineage progression is orchestrated by transcriptional networks composed of intrinsic gene regulatory programmes. While key regulators such as OLIG2, SOX10 and MYRF have been extensively characterised^9–13^, the full complement of transcription factors (TFs) controlling early differentiation remains incompletely defined. In particular, the intrinsic mechanisms governing the OPC-to-NFOL transition are not fully understood.

*Myt1*, one of the first transcriptional factors described to have roles in OL lineage regulation^14^, is a member of a three-gene family. Here, we report expression and function of *St18/Myt3*, encoding a zinc-finger transcription factor, as marker and regulator of oligodendrocyte differentiation. By combining snRNA-seq and snATAC-seq analyses with *in vitro* and *in vivo* functional assays, we show that *St18* is selectively upregulated at the onset of differentiation, promotes oligodendrocyte maturation, and is controlled by oligodendroglial TFs including SOX10, MYRF, and KLF9/13. Interestingly, phylogenetic and synteny analyses indicate that *St18* arose in the last common ancestor of jawed vertebrates, coinciding with the emergence of myelinating oligodendrocytes. Together, these findings identify *St18* as a marker of NFOLs and OLs as well as a transcription factor with activities in oligodendrocyte differentiation.

## Results

### *St18* is first expressed in newly formed oligodendrocytes (NFOLs).

To identify TFs regulating the transition from OPCs to NFOLs, we reanalyzed a snRNA-seq dataset of the remyelinating mouse spinal cord^15^. OPC and NFOL nuclei from the naïve spinal cord and day 5 post lysolecithin injection were subsetted and reclustered, resulting in clear segregation of populations according to differentiation stage (Fig. 1A–C). Cluster identities were validated by the expression of canonical OPC markers (e.g. *Pdgfrα*, *Cspg4*), NFOL markers (e.g. *Bcas1*, *Enpp6*), and myelin-associated genes (e.g. *Plp1*, *Myrf*) (Fig. 1D). Differential gene expression analysis revealed a coordinated downregulation of OPC-associated genes (e.g. *Pdgfrα*, *Cspg4/5*, *Pcdh15*) and upregulation of NFOL and myelin-related genes, including *Enpp6*, *Bmp4*, *Bcas1*, *Qk*, and *Plp1*.

Among the top differentially expressed genes, *St18*, encoding a C2H2-type zinc finger transcription factor, was prominently upregulated in NFOLs (Fig. 1E). To further investigate its temporal dynamics, we constructed a pseudotime trajectory using reclustered OPC and NFOL populations (Fig. 1F). This analysis revealed a progressive transcriptional transition marked by early downregulation of *Pdgfrα* followed by induction of *Enpp6* at later stages. Notably, *St18* upregulation preceded *Enpp6*, suggesting that it may act as an early regulator of NFOL differentiation (Fig. 1G). We next assessed *St18* expression across CNS cell types (Fig. 1H). *St18* expression was largely restricted to the oligodendroglial lineage, with minimal expression in OPCs and increased expression in NFOLs and mature oligodendrocytes. Its expression pattern was distinct from *Pdgfrα* and overlapped with differentiation-associated markers such as *Enpp6* and *Ermn*, further supporting its association with differentiating oligodendrocytes (Fig. 1I–J). The mouse oligodendroglial expression profile of *St18* was largely conserved in a human MS snRNA-seq dataset^16^, with expression also detected in phagocytes, potentially highlighting a role of *ST18* in human myeloid cells (Supplementary Fig. S1). Collectively, these findings reveal a conserved pattern of *St18* upregulation during oligodendrocyte differentiation in both mouse and human, highlighting its potential role as an early regulator of NFOL lineage progression.

### *St18* is a central hub of the NFOL transcriptional program.

To further define the regulatory programs associated with NFOL states, we performed high-dimensional weighted gene co-expression network analysis (hdWGCNA) on reclustered OPC and NFOL nuclei. Unlike differential expression analysis, which identifies genes based on expression changes between groups, hdWGCNA infers gene regulatory architecture by identifying modules of co-expressed genes and prioritizing hub genes based on network connectivity. This analysis identified nine gene modules across OPC and NFOL populations (Fig. 2A, Supplementary Fig. S2A). Module eigengene expression profiles revealed distinct enrichment patterns across subclusters, and the top five hub genes per module were ranked according to their module association (kME) values (Fig. 2B, Supplementary Fig. S2B). Among these modules, the turquoise and red modules were strongly enriched in NFOL clusters (Fig. 2C). Notably, the turquoise module emerged as the most robust pan-NFOL gene module, encompassing canonical NFOL markers, including *Enpp6* and *Bcas1* (Fig. 2D, Supplementary Fig. S2C). Strikingly, *St18* was identified as the top-ranked hub gene within this module, followed by *Enpp6* and the long non-coding RNA 9540059O14Rik, which was also highlighted in differential expression analysis (Fig. 2D, Fig. 1E). These findings indicate that the turquoise module represents a core transcriptional program underlying NFOL differentiation, with *St18* positioned as a central regulatory node.

To validate these observations *in vivo*, we performed single-molecule fluorescence in situ hybridization (smFISH) by RNAscope on full sagittal P7 mouse brain sections (Fig. 2E). We examined *St18* expression across oligodendrocyte lineage stages using established markers, including *Pdgfra* (OPCs), *Enpp6* (NFOLs), and *Ermn* (mature oligodendrocytes). Consistent with the single-nucleus RNA-sequencing data, *St18* transcripts were predominantly detected in *Enpp6*- and *Ermn*-positive cells within white matter–enriched regions such as the pons, corpus callosum, and cerebellar white matter (Fig. 2F). Together, these findings establish *St18* expression during oligodendrocyte differentiation, with enriched expression in NFOLs and mature oligodendrocytes and a clear distinction from OPCs (Fig. 2G). Its strong upregulation and connectivity within the NFOL co-expression module, coupled with its early induction preceding canonical markers such as *Bcas1* and *Enpp6*, supports a role for *St18* in the initiation and progression of oligodendrocyte differentiation.

### *St18* promotes oligodendrocyte differentiation *in vitro* and regulates the timing of MBP expression *in vivo*.

We next investigated roles for *St18* in oligodendrocyte differentiation using primary OPCs isolated from postnatal day 5 (P5) rat brains with gain-of-function using a lentiviral construct encoding rat *St18* or an mCherry control. Primary OPCs were transduced one day after plating and allowed to express the transgene for two days prior to differentiation (Fig. 3A–B). Overexpression (OE) of *St18* was confirmed by HA-tag immunoreactivity in transduced oligodendrocytes (mCherry+ Olig2+ cells), which localized to the nucleus, consistent with its function as a transcription factor (Supplementary Fig. S3A). Moreover, *St18* transcript levels were significantly increased in OE cultures compared to controls (Supplementary Fig. S3B). Following induction of differentiation with triiodothyronine (T3), oligodendrocyte differentiation was assessed at day 3 and day 5 by immunostaining for canonical myelin markers, including myelin basic protein (MBP) and 2’,3’-cyclic nucleotide 3’-phosphodiesterase (CNP). Quantitative analysis revealed a significant increase in the proportion of MBP^+^ and CNP^+^ cells co-expressing OLIG2^+^/mCherry^+^ with St18 OE compared to controls, indicating accelerated and enhanced differentiation (Figs. 3C, D). These findings demonstrate that *St18* overexpression promotes oligodendrocyte differentiation in primary OPCs.

To investigate the role of *St18 in vivo*, we generated a conditional knockout (cKO) mouse model by crossing a *Sox10-cre* driver line^17^ with a floxed *St18* allele^18^, enabling lineage-specific ablation in oligodendrocytes (Fig. 3E, Supplementary Fig. S4A-D). At postnatal day 0 (P0), immunohistochemical (IHC) analysis revealed a significant reduction in MBP-positive area in the spinal cords of mutant mice compared to controls (Fig. 3F). This deficit in myelination persisted at P7, with reduced MBP intensity in the cerebellar white matter of ST18 cKO mice compared with controls (Fig. 3G). To determine whether delayed MBP expression impacted axonal domain organization, we quantified nodes of Ranvier by co-immunostaining of MBP and CASPR, labelling myelin sheaths and paranodal junctions, respectively. *St18*-deficient mice exhibited a significant reduction in paranodal structures, suggesting delayed assembly or defective organisation of the nodes of Ranvier (Fig. 3H). Despite these early deficits, by P14 MBP staining intensity and distribution in both cortex and cerebellar white matter were similar in cKO animals and controls, suggesting that St18 is primarily required for the timely onset of myelination rather than for long-term maintenance of the myelinated state (Supplementary Fig. S4E).

### A Sox10-dependent regulatory network controls *St18* activation during oligodendrocyte differentiation.

We next investigated *St18* upstream regulatory mechanisms during lineage progression, aiming to identify candidate *cis*-acting DNA regulatory elements (CREs) and TFs governing *St18* activation. To this end, we analysed single-nucleus assay for transposase-accessible chromatin using sequencing (snATAC-seq) data from mouse spinal cord remyelination^15^ and matched timepoints to those in the snRNA-seq dataset (Fig. 1), enabling high-resolution profiling of chromatin accessibility across the *St18* locus during oligodendrocyte differentiation. Chromatin accessibility at the *St18* locus increased progressively along the differentiation trajectory, with prominent accessible regions emerging at the transcriptional start site (TSS), as well as within intragenic and flanking regions (Figs. 4A, B). This pattern appeared consistent with the observed transcriptional upregulation of *St18* in NFOLs and mature oligodendrocytes. To identify candidate regulatory elements driving *St18* expression at early stages of differentiation, we reclustered OPC and NFOL nuclei from naïve and 5 days post-lesion (5 dpl) samples (Fig. 4C) and applied Cicero to infer co-accessible chromatin regions. This analysis identified multiple candidate CREs linked to the *St18* TSS, representing putative enhancer elements activated during the OPC-to-NFOL transition (Fig. 4D).

Motif enrichment analysis of these candidate regions revealed significant enrichment for binding motifs of several TFs (Fig. 4E). Among these, motifs for CTCF were detected, consistent with a potential role in higher-order chromatin organization. Notably, strong enrichment was also observed for SOX transcription factor motifs including SOX10, a central regulator of oligodendrocyte lineage; additionally, we analyzed a SOX10 ChIP–seq dataset from postnatal rat spinal cords^19^. This identified multiple high-confidence SOX10 binding sites in proximity to the *St18* locus, including regions located −114 kb, −70 kb, −22 kb upstream and +115 kb, +225 kb, and +522 kb downstream of the TSS (Fig. 4F). Among these, the −114 kb and +115 kb regions were prioritized for further study due to the presence of evolutionarily conserved regions (ECRs), suggesting functional regulatory relevance (Fig. 5A). To test whether these candidate elements are responsive to oligodendroglial TFs, we performed luciferase reporter assays in HEK293T cells. The −114 kb and +115 kb regions were co-transfected with expression constructs encoding *Sox10*, *Myrf*, and *Klf9* or *Klf13*. While *Sox10* and *Myrf* were insufficient to induce reporter activity, co-expression with *Klf9* and its paralog *Klf13* strongly activated the luciferase reporter, with maximal activation observed when all three factors were present (Figs. 5B, C). Moreover, to validate the role of *Sox10* in regulating *St18* expression *in vivo*, we performed smFISH in the corpus callosum of P7 control and *Olig2-cre X Sox10^fl/fl^* conditional knockout mice. As shown (Figs. 5D, E), loss of *Sox10* resulted in a significant reduction in *St18* transcripts within *Enpp6*^+^ differentiating oligodendrocytes, confirming that *Sox10* is required for *St18* expression during differentiation *in vivo*.

Finally, we explored whether *St18* may be integrated into previously described regulatory pathways controlling oligodendrocyte differentiation. Recent work^20^ has identified a retrotransposon-derived element, *RetroMyelin (RNLTR12-int)*, which binds to SOX10 and promotes activation of myelin-associated genes. Consistent with this model, analysis of bulk RNA-sequencing data from differentiating OPCs revealed that knockdown of *RNLTR12-int* led to reduced *St18* expression (Fig. 5F), suggesting that *St18* may act downstream of SOX10-dependent *RNLTR12-int*-mediated regulatory mechanisms. Together, these findings define a cis-regulatory architecture underlying *St18* activation during oligodendrocyte differentiation and identify a cooperative transcriptional network involving *Sox10*, *Myrf*, and *Klf9/13*. This regulatory framework positions *St18* downstream of these key lineage determinants and supports its role as an early effector of the oligodendrocyte differentiation program.

### Evolutionary emergence of *St18 (Myt3)* coincides with the onset of myelination in jawed fishes.

*RNLTR12-int* is a retrotransposon-derived regulatory element that enables SOX10 binding to myelin gene promoters such as *Mbp*^20^. Notably, *RNLTR12-int* emerges in the genome concomitantly with the appearance of vertebrate myelination at the transition from jawless (e.g., lamprey) to jawed fishes (e.g., elephant shark). Given evidence for *St18* regulation by *RNLTR12-int*, we next considered *St18* gene ontogeny. While core components of the oligodendrocyte gene regulatory network (GRN), including *Sox10* and *Myrf*, are essential for oligodendrocyte identity, these factors predate the emergence of myelination (Fig. 6E). This suggests that additional regulatory genes and elements were required to couple a pre-existing GRN to the acquisition of myelinating function. To investigate how this system was assembled over evolutionary time, we integrated single-cell transcriptomic data with comparative genomics to generate an evolutionary framework of oligodendrocyte differentiation. We first selected genes differentially expressed between OPCs and NFOLs in both mouse and human single-cell datasets and mapped their evolutionary origins across a species phylogeny (Fig. 6A). This phylostratigraphic analysis revealed a subset of genes that emerged at the onset of myelination, including 384 and 134 genes in mouse and human, respectively, that first appear in *Gnathostomata* (jawed fishes) (Figs. 6B, C, Supplementary Table S1). Extending this approach across species enabled the identification of 54 conserved genes associated with myelination (Fig. 6D). Notably, *St18* was among these conserved genes together with myelin genes such as MBP, MAG, MOG and CLDN11. Consistently, the evolutionary emergence of *St18* coincided with vertebrate myelination, while its expression correlated with myelin gene expression across species (Fig. 6E).

We next investigated the evolutionary origin of *St18*, reasoning that gene innovation may underlie the emergence of new cellular states. *St18* belongs to the *MYT* family of zinc finger TFs, which also includes *Myt1* and *Myt1l*. Analysis of single-cell data revealed distinct expression patterns across the oligodendroglial lineage: *Myt1* is predominantly expressed in OPCs, *St18* is enriched in NFOLs and myelinating oligodendrocytes, and *Myt1l* is expressed in other neural cell types (Fig. 6F, Supplementary S5), suggesting functional diversification within this family. Consistently, pseudotime analysis of the OPC-to-NFOL transition revealed reciprocal expression dynamics of *Myt1* and *St18*, with *Myt1* progressively declining as St18 is induced during oligodendrocyte differentiation, consistent with functional diversification of these paralogous transcription factors across lineage progression (Fig. 6G).

Phylogenetic analysis of MYT family proteins resolved four distinct clades corresponding to *Myt1*, *Myt1l*, and *St18* in gnathostomes, as well as more ancestral agnathan outgroups (Fig. 6H). Within gnathostomes, *Myt1* occupies a basal position relative to *Myt1l* and *St18*, supporting a model in which *Myt1* represents the ancestral gene that underwent duplication events to give rise to *Myt1l* and *St18*. The presence of Myt1l orthologs in agnathans indicates that the *Myt1*-to-*Myt1l* duplication predates the divergence of jawless vertebrates, whereas *Myt*1-to-*St18* duplication likely emerged in the gnathostome lineage, coinciding with the evolution of myelin (Fig. 6H).

To further resolve the origin of *St18*, we performed microsynteny analysis across representative species. Comparison of genomic loci in lamprey (*Petromyzon marinus*), a jawless vertebrate, and elephant shark (*Callorhinchus milii*), an ancient and slow-evolving jawed vertebrate^21^, revealed that the lamprey *Myt1* locus shares conserved gene order and sequence similarity with both *Myt1* and *St18* loci in elephant shark (Fig. 6I). This conserved microsynteny supports the hypothesis that *St18* arose through duplication of an ancestral *Myt1* locus in early gnathostomes. In contrast, *Myt1l* displayed conserved synteny only with itself, consistent with an independent duplication event from *St18* (Fig. 6J). Taken together, these findings support a model in which *St18* emerged through duplication and divergence from an ancestral *Myt1* gene at the onset of vertebrate myelination. This evolutionary innovation coincides with the transition from ancestral glia to differentiating and myelinating oligodendrocytes, suggesting that *St18* contributed to the acquisition of myelin.

## Discussion

We analysed expression, regulation, function and evolutionary features of *St18*, encoding a zinc finger transcription factor. While ours is the first report of detailed St18 expression, it is notable that its expression is commonly used in sc/snRNA seq. analyses to identify the oligodendrocyte lineage^22,23^.

### St18 promotes the timely progression of oligodendrocyte differentiation.

Differentiation of OPCs into myelinating oligodendrocytes requires a coordinated transition from a proliferative progenitor state toward a highly specialized postmitotic identity. We report *St18* as a component of the oligodendrocyte differentiation program that becomes activated during the OPC-to-NFOL transition, commencing prior to canonical NFOL markers such as *Enpp6* and *Bcas1*. This timing is notable because the OPC-to-NFOL transition involves extensive chromatin remodeling, cell-cycle exit, cytoskeletal reorganization and metabolic adaptation^24,25^. While *St18* continued to be expressed in mature oligodendrocytes, *St18* deficiency did not abolish oligodendrocyte differentiation but instead delayed the onset of MBP expression and paranodal assembly. This phenotype supports a model in which *St18* acts to stabilize or promote differentiation trajectories rather than specify oligodendrocyte identity itself. Such regulators may be particularly important during transitional cellular states to finely tune the timing of differentiation. The transient developmental phenotype observed following *St18* deletion further suggests that compensatory mechanisms or partially redundant transcriptional pathways may eventually overcome the absence of *St18* during later stages of myelination. Whether *St18* contributes to remyelination following injury or in demyelinating disease contexts remains an important question for future studies.

### Upstream regulation of St18 by SOX10, MYRF and KLF9/13.

Chromatin accessibility analyses identified multiple candidate CREs associated with the *St18* locus that become activated during oligodendrocyte differentiation. Enrichment of SOX-family motifs together with SOX10 occupancy at these regions supports a model in which *St18* is directly controlled by core oligodendroglial regulators. The cooperative activation of *St18*-associated enhancers by SOX10, MYRF and KLF9/13 is particularly informative mechanistically. SOX10 and MYRF are central regulators of oligodendrocyte identity and myelin gene expression^9,13^, whereas KLF9 has been linked to thyroid hormone–mediated differentiation and timing of oligodendrocyte maturation^26,27^. The inability of SOX10 or MYRF alone to strongly activate reporter activity suggests that *St18* expression may depend on combinatorial TF activity that emerges specifically during transitional differentiation states. Such combinatorial regulation may provide temporal precision, ensuring that *St18* is activated only after progenitors initiate differentiation.

### Evolutionary appearance of *St18/Myt3* at the transition of jawless to jawed fishes.

Our findings provide insight into how oligodendrocyte differentiation programs may have evolved. Several major regulators of oligodendrocyte identity predate vertebrate myelination, implying that the emergence of myelin required the integration of novel regulatory components into pre-existing glial transcriptional networks aligning with recent work^20^. The identification of *St18* as a gnathostome-associated gene enriched in differentiating oligodendrocytes supports this model. Phylogenetic and syntenic analyses suggest that *St18* arose through duplication and divergence of an ancestral *Myt1* locus during early vertebrate evolution. Notably, *Myt1* and *St18* now display distinct expression domains across the oligodendroglial lineage^28^, with *Myt1* enriched in OPCs and *St18* enriched in differentiating oligodendrocytes. *Myt1* has previously been implicated in maintaining neural progenitor identity and regulating progenitor differentiation programs through transcriptional repression mechanisms^29^, and is a key marker/regulator of OPCs^30^. The emergence of *St18* from an ancestral *Myt1* locus may therefore reflect functional specialization toward transitional oligodendrocyte states to enable myelination. This neofunctionalization following duplication may have enabled increasingly specialized regulation of differentiation states. More broadly, these findings support the idea that evolutionary innovation in the CNS may involve not only the emergence of novel cell types, but also the refinement of transitional cellular states and their associated regulatory programs. The emergence of *St18* may therefore represent part of a broader evolutionary process through which ancestral glial regulatory networks acquired additional layers of temporal and transcriptional control necessary for efficient vertebrate myelination.

## Methods

### Single-nucleus transcriptomic analysis.

snRNA-seq data from remyelinating mouse spinal cord were generated and preprocessed as described in^15^. The processed dataset was provided as a Seurat object containing log-normalised expression values and cell-level metadata and was imported into R for downstream analysis using Seurat (v5.2.1).

To refine oligodendrocyte lineage populations, cells annotated as OPCs from young animals at the naïve and 5 dpl timepoints were subsetted for reclustering. Highly variable genes were identified using the variance-stabilising transformation (VST) method implemented in FindVariableFeatures, retaining the top 2,000 variable features. Gene expression values were then z-score scaled across all genes using ScaleData. Dimensionality reduction was performed using principal component analysis (PCA) on the set of highly variable genes. The number of informative principal components was evaluated using JackStraw permutation testing (JackStraw, 100 replicates) and ElbowPlot inspection. The first 12 principal components were selected for downstream analysis. Cell–cell similarity was computed using a shared nearest neighbour (SNN) graph constructed with FindNeighbors using the first 12 principal components. Clustering was performed using the Louvain community detection algorithm implemented in FindClusters with a resolution parameter of 0.1. Low-dimensional embeddings were generated using Uniform Manifold Approximation and Projection (UMAP) (RunUMAP) and t-distributed stochastic neighbour embedding (t-SNE) (RunTSNE) based on the same set of principal components to visualise transcriptional relationships between cells. Subclusters corresponding to OPCs and NFOLs were annotated based on canonical marker gene expression, including *Pdgfra*, *Cspg4*, *Gpr17*, *Enpp6*, *Bcas1*, *Myrf*, and *Mbp*. Marker gene expression patterns were visualised using heatmaps generated from both z-score scaled expression values and log-normalised expression values. Following identification of *St18* as a gene of interest, its expression was examined across the full snRNA-seq dataset. The complete Seurat object containing all annotated cell populations was used to visualise *St18* expression across cell types and experimental conditions. Gene expression patterns were visualised using UMAP feature plots and violin plots generated with Seurat to assess the distribution and relative enrichment of *St18* across oligodendrocyte lineage states and other CNS cell populations.

### Pseudotime trajectory and trajectory-associated gene analysis.

Trajectory inference was performed using Monocle3 (v1.0.0) to reconstruct transcriptional progression from OPC to NFOL states. The reclustered Seurat object containing young naïve and 5 dpl OPC and NFOL nuclei was converted to a Monocle3 cell_data_set object using SeuratWrappers. Size factors were estimated using estimate_size_factors, and gene annotations were transferred by assigning gene names to the gene_short_name field. Cells were clustered using cluster_cells based on the existing UMAP embedding, and a principal trajectory graph was learned using learn_graph. The trajectory root was manually defined by selecting cells corresponding to the earliest OPC state in UMAP space, and pseudotime values were assigned to each nucleus using order_cells. Genes associated with pseudotime progression were identified using graph_test, which computes Moran’s I statistic to detect genes whose expression varies along the inferred trajectory. Genes with the strongest pseudotime-associated signal were ranked by Moran’s I. The top variable genes were selected for downstream trajectory differential expression analysis. To visualise gene expression dynamics along the trajectory, expression values were modelled as a smooth function of pseudotime using the model:

y~sm.ns(pseudospace,df=3)

where gene expression is fitted using a natural spline of pseudotime. Expression dynamics were visualised using plot_genes_in_pseudotime to examine continuous transcriptional changes across the OPC to NFOL differentiation trajectory.

### High dimensional weighted gene co-expression network analysis.

High-dimensional weighted gene co-expression network analysis (hdWGCNA) was performed using the hdWGCNA R package (v0.4.03) to identify gene co-expression modules associated with oligodendrocyte lineage states. Analyses were conducted on a Seurat object containing reclustered young naïve and 5 dpl OPC and NFOL nuclei. To reduce sparsity inherent to single-cell transcriptomic data, metacells were constructed using K-nearest neighbour aggregation implemented in hdWGCNA. Metacells were generated separately for each sample and transcriptional subcluster using K = 20 nearest neighbours, while restricting overlap between metacells to ≤10 shared cells to minimise oversmoothing. Metacell-level expression matrices were scaled and used as input for network construction. Pairwise gene–gene correlations were computed across metacells to generate a signed correlation matrix, which was transformed into an adjacency matrix using a soft-thresholding power selected based on the scale-free topology fit criterion. The adjacency matrix was subsequently converted into a topological overlap matrix (TOM) to quantify network interconnectedness between genes. Modules of co-expressed genes were identified by hierarchical clustering based on TOM dissimilarity (1–TOM) followed by dynamic tree cutting. For each module, the module eigengene (ME), defined as the first principal component of the expression matrix of module genes, was calculated to represent overall module activity. Module eigengenes were computed both with and without Harmony batch correction to account for potential sample-level effects. Gene connectivity within modules was quantified using kME, defined as the correlation between individual gene expression profiles and the corresponding ME. Genes with the highest kME values were considered hub genes, and the top 25 hub genes per module were selected for downstream analyses. To assess module activity at single-cell resolution, hub gene sets were scored using the UCell method, generating per-cell module enrichment scores. Module activity was visualised across cell states using UMAP projections, dot plots, and radar plots.

### Single nucleus ATAC-seq analysis.

Single-nucleus ATAC-seq (snATAC-seq) data from remyelinating mouse spinal cord were generated and preprocessed as described in^15^. Briefly, sequencing reads were aligned, fragments were generated, and accessible chromatin peaks were identified as part of the upstream processing pipeline. The processed dataset was provided as a Seurat object containing chromatin accessibility counts and cell-level annotations and was imported into R for downstream analysis using Seurat and Signac. Chromatin accessibility across annotated cell populations was visualised using UMAP embeddings stored in the Seurat object. Cell populations were displayed using DimPlot grouped by annotated cell type. Accessibility associated with specific genes was visualised using FeaturePlot to project gene activity scores onto the UMAP embedding. To examine accessibility patterns within the oligodendrocyte lineage, cells annotated as OPCs were subsetted from the full dataset and restricted to young animals at the naïve and 5 dpl timepoints. Accessible peak features were retained using FindTopFeatures with min.cutoff = ’q0’, and dimensionality reduction was performed using latent semantic indexing (LSI) implemented in RunSVD. The relationship between sequencing depth and LSI components was evaluated using DepthCor, and the first LSI component was excluded from downstream analyses due to its association with sequencing depth. Cell–cell similarity was computed using FindNeighbors based on LSI dimensions 2–8, and clustering was performed using FindClusters with algorithm = 3. Low-dimensional embeddings were generated using RunUMAP with LSI dimensions 2–8 to visualise chromatin accessibility patterns across oligodendroglial populations.

Chromatin accessibility at loci of interest was examined using CoveragePlot, which visualises fragment coverage across genomic regions. Accessibility profiles were examined at the St18 locus (chr1:6312485–7045504) to assess regulatory accessibility across OPC and NFOL populations.

### Linking *St18* to candidate cis-regulatory elements.

Co-accessible chromatin regions were identified using the Cicero framework to infer candidate cis-regulatory elements associated with genes of interest. snATAC-seq data restricted to OPC from young naïve and 5 dpl samples were converted to a cell_data_set object using SeuratWrappers. Sparse chromatin accessibility matrices were extracted and converted into Cicero input format containing peak, cell, and count information. Accessibility counts were binarised using make_atac_cds(binarize = TRUE) to generate the Cicero cell dataset. Cells were processed using detectGenes and estimateSizeFactors, and dimensionality reduction was performed using reduceDimension with t-SNE (num_dim = 6, max_components = 2). The resulting embedding was used to construct a Cicero object using make_cicero_cds, and chromatin co-accessibility scores were computed using run_cicero with chromosome sizes derived from the mm10 reference genome. This algorithm estimates correlations in accessibility between genomic regions while accounting for genomic distance. Gene annotations were obtained from the Ensembl GRCm38/mm10 annotation (release 102) and processed to generate gene models compatible with Cicero visualisation. Only exonic features were retained for gene model construction, and the longest transcript model was used when collapsing transcripts. Co-accessible regulatory interactions were visualised using plot_connections, focusing on the St18 locus (chr1:6312485–7045504). Connections were filtered using a co-accessibility cutoff of 0.25 to highlight high-confidence regulatory interactions.

### Motif enrichment analysis.

Hypergeometric Optimization of Motif EnRichment (HOMER, v5.1) was employed to discover enrichment of putative DNA binding motifs. Genomic loci of human ECRs and mouse cCREs were compiled as BED files, and findMotifsGenome.pl was run to discover enriched motifs 200bp upstream and downstream of these sites. Human ECRs and mouse cCREs were screened against hg19 and mm10 genomic assemblies respectively:

- findMotifsGenome.pl [BED file] mm10 [output directory] -size 200 -mask
- findMotifsGenome.pl [BED file] hg19 [output directory] -size 200 -mask

Motif enrichment p-values were calculated using HOMER’s default statistical framework. Briefly, HOMER compares the frequency of motif occurrences in the input genomic regions (target set) versus a GC-matched set of randomly selected background regions from the genome. The significance of motif enrichment is assessed using a binomial test, which models the number of motif-containing sequences in the target set relative to the background expectation. The binomial test is appropriate when the background set is large and randomly sampled, as was the case in this analysis.

### SOX10 ChIP-seq analysis.

SOX10 peak coordinates were extracted from^19^ (GSE64703) by using HOMER for peak calling. The resulting .bed files were uploaded to the UCSC web interface (https://genome.ucsc.edu/cgi-bin/hgLiftOver) and rat genomic coordinates were converted into mouse genomic coordinates aligning to the mm10 genome assembly. The resulting mouse .bed file was then imported and visualised in IGV.

### Phylostratigraphic analysis.

Differentially expressed gene lists between OPCs and NFOLs from human^15^ and mouse^14^ snRNA-seq were filtered (padj < 0.05, |avg_log2FC| > 0.5), and gene symbols resolved to Ensembl IDs via the Ensembl REST API (unresolved symbols excluded). For each gene, the corresponding Ensembl Compara gene tree (vertebrates clade, TreeBeST-based, reconciled against the species tree^28^) was retrieved via the genetree/member/id endpoint (phyloXML, sequence=none) and cached locally. Each tree was parsed for NCBI taxonomy, node depth, branch length, and duplication/speciation annotation at every internal node. A gene’s phylostratum was assigned as the most taxonomically derived node matching a fixed, ordered reference taxonomy from LUCA to the focal species (28 ranks; full list in code/config.json, ancestor_taxonomy_order), with ties broken by minimum node depth and unmatched trees falling back to the root (flagged via match_mode). Assignments were validated against literature-established origins for five well-characterised genes.

### Phylogenetic analysis.

Canonical protein sequences of the longest isoform of Myt1, Myt1l and St18 from different species were collected from UniProt as FASTA files which were used for multiple sequence alignment (MSA) using MUltiple Sequence Comparison by Log-Expectation^31^ with default parameters. Alignment results were collected as CLUSTAL format. The maximum likelihood trees were constructed with default parameters using IQ-TREE with ultrafast bootstrapping analysis with 1000 bootstrapping alignments^32^. Consensus tree files containing bootstrapping values were visualised in interactive tree of life (iTOL).

### Synteny analysis.

Microsynteny analysis using the jcvi.compara module of the Python implementation of MCScanX was performed. 20 genes upstream and downstream of *Myt1*, *Myt1l* and *St18* on lamprey and elephant shark scaffolds and human chromosomes were used for analysis. C scores of 0.99 were used for lamprey and elephant shark *St18* and *Myt1*, 0.70 for lamprey and elephant shark *St18* and *Myt1l*, and 0.99 for human *St18* and *Myt1*. Microsyntenic linkages were visualised using jcvi.graphics.synteny.

### Primary rat OPC isolation.

OPCs were isolated from unsexed P5 rat brains largely as previously described^33,34^. Fresh P5 Wistar/Han rat heads in cold Hibernate^™^-A Medium (Thermo Scientific, A1247501) were obtained from Charles River Laboratories (Margate, UK) following cervical dislocation. Whole brains were dissected, meninges removed, and tissue homogenised with a sterile scalpel on ice. Tissue was washed in Ca^2+^/Mg^2+^-free HBSS (HBSS −/−; Thermo Scientific, 14170112) and digested in papain solution (34 U/mL papain, Worthington, LS003126; 20 μg/mL DNase I, Sigma, 10104159001 in Hibernate^™^-A) for 20 min at 37 °C with shaking (55 rpm). After centrifugation (200 g, 5 min, RT), pellets were triturated in neutralising solution (2% B27, Thermo Scientific, 17504044; 2 mM sodium pyruvate, Sigma-Aldrich, S8636; 20 μg/mL DNase I in Hibernate^™^-A) to generate a single-cell suspension. The supernatant was filtered through a pre-wet 70 μm strainer into isotonic Percoll (90% Percoll, Sigma-Aldrich, GE17-5445-01; 10% 10X PBS, Thermo Fisher, 70011044) and centrifuged (800 g, 20 min, RT, no brake; final 22% Percoll). Debris was aspirated, and cells were washed in HBSS −/− followed by red blood cell lysis (Sigma-Aldrich, R7757). Myeloid cells were depleted using anti-rat CD11b/c magnetic microbeads (Miltenyi Biotec, 130-105-643) diluted in Miltenyi wash buffer (MWB; PBS pH 7.3 containing 2 mM EDTA, Thermo Scientific, 15575020; 2 mM sodium pyruvate; and 0.5% BSA, Sigma-Aldrich, A8327) supplemented with 10 ng/mL human recombinant insulin (Sigma-Aldrich, I9278), according to the manufacturer’s instructions using LS MACS columns (Miltenyi Biotec, 130-042-401). OPCs were enriched by A2B5 selection using mouse anti-A2B5 antibody (0.125 μL per brain; Sigma, MAB312) followed by anti-mouse IgM microbeads (Miltenyi Biotec, 130-047-302) and LS column separation. Purified OPCs were eluted in OPC basal medium containing N-acetyl cysteine (60 μg/mL; Sigma-Aldrich, A8199), human recombinant insulin (10 μg/mL), sodium pyruvate (1 mM), apo-transferrin (50 μg/mL; Sigma-Aldrich, T1147), putrescine (16.1 μg/mL; Sigma-Aldrich, P5780), sodium selenite (40 ng/mL; Sigma-Aldrich, S5261), progesterone (60 ng/mL; Sigma-Aldrich, P8783), and BSA (330 μg/mL; Sigma-Aldrich, A8327), supplemented with bFGF (20 ng/mL; PeproTech, 100-47), PDGF-AA (20 ng/mL; PeproTech, 100-13A), and 1% Pen/Strep (Thermo Scientific, 15140122).

### OPC culture and differentiation.

Isolated OPCs were seeded at 13,000 cells/cm^2^ onto μ-Plate 24-well plates (Ibidi, 82426) pre-coated with poly-D-lysine (50 μg/mL; Sigma, P6407) and laminin (20 μg/mL; Bio-Techne, 3446-005-01). OPCs were left to recover overnight in OPC basal media supplemented with 20 ng/mL human bFGF, 20 ng/mL human PDGFα and 1% Pen/Strep and incubated at 37 °C, 5% CO2 and 5% O2. The following day the media was completely changed to fresh OPC basal media with 10 ng/ml bFGF and PDGFα without Pen/Strep to remove dead cells and promote cell proliferation and incubated for 48 hours. To promote differentiation, the media was completely changed to OPC basal media supplemented with 40 ng/ml T3 (Sigma, T6397) and cultured for 3 to 5 days. Media was changed every 48 hours for all experiments to ensure regular exposure to fresh metabolites and growth factors.

### Lentivirus and transduction.

To produce lentiviral particles, custom transfer plasmids were obtained from Vectorbuilder: pLV[Exp]-CBh>{rSt18:HA:T2A:mCherry} (a.k.a ST18-OE, VB240324-1065qdn) and pLV[Exp]-CBh>{mCherry} (a.k.a Control, VB240324-1067kqz); and helper plasmids were obtained from Addgene: VSVG (Addgene, Plasmid #14888) and psPAX2 (Addgene, Plasmid #12260). HEK 293T (ATCC, CRL-3216) were cultured in PDL-coated 15 cm dishes in HEK media DMEM/F12 (Thermo Scientific, 21331020) supplemented with 10% FBS (Thermo Scientific, A5256701) at 37°C until 70% confluence 1 day before transfection. For one 15 cm cell culture dish, 4.5 μg of either ST18-OE or Control transfer plasmid, VSVG and psPAX2 were added to 2.25 ml Opti-MEM^™^ with reduced serum media (Thermo Fisher, 31985070), and 54 μl of Lipofectamine 2000^™^ Transfection Reagent (Thermo Fisher, 11668019) was added to 2.25 ml Opti-MEM^™^ with reduced serum media in two separate 15 ml falcon tubes. After incubation (5 min, RT), the two falcon tubes were combined and incubated (20 min, RT) to create DNA/lipid complexes which were added directly to the HEK 293T cell culture dish and incubated overnight. The media was then completely changed to fresh OPC basal media without growth factors or antibiotics and incubated for 24 hours. The cell culture supernatant was collected, centrifuged (10 min, 500 x g, RT) filtered through a 0.45 μm filter, and stored at −80 °C. For primary OPC transduction, lentivirus-enriched OPC basal media was diluted in fresh OPC basal media 1:10 followed by addition of 20 ng/ml bFGF and PDGFα. Cell media was completely changed 1 day after isolation, and lentiviral infection was allowed to continue for 48 hours. The media was fully changed to OPC basal media supplemented with 40 ng/ml T3 to enable differentiation.

### Immunocytochemistry (ICC).

Culture media was removed and cells were fixed with 4% PFA (Sigma, #158127) for 15 minutes at room temperature followed by three washes with PBS. The fixed cells were either stored at 4°C before use in PBS or directly blocked and permeabilized in blocking buffer (0.1% Triton X-100 (Sigma, #T8787), 5% normal donkey serum (NDS, Abcam, ab7475) in PBS) for 1 hour at room temperature. Primary antibodies (Mouse anti-HA-tag (Cell Signaling, C29F4), Chicken anti-mCherry (Abcam, ab205402), Rabbit anti-mCherry (Abcam, ab167453), Mouse anti-OLIG2 (Sigma-Aldrich, MABN50), Goat anti-OLIG2 (R&D Systems, AF2418), Rat anti-MBP (Bio-Rad, Aa82–87), Mouse anti-CNPase (Sigma-Aldrich, C5922)) were diluted in blocking buffer solution and incubated on the fixed cells overnight at 4 °C. The wells were washed three times with PBS before addition of secondary Alexa Fluor antibodies (Thermofisher) for 1 hour at room temperature and protected from light. The wells were washed three times with PBS and 10 μg/ml Hoechst stain in PBS and incubated (20 min, RT) protected from light, followed by three PBS washes. Confocal images were taken with Operetta (PerkinElmer). Further image processing was performed using Fiji and quantification was performed using Harmony (PerkinElmer). Data was processed using Rstudio (v4.4.2).

### Animal husbandry.

*Sox10-cre* transgenic mice^17^ were provided by William Richardson, and St18^fl/fl^ mice^18^ were provided by the Gu Laboratory (Vanderbilt University, TN, USA). Conditional knockout animals (*Sox10-cre; St18^fl/fl^*) were bred and maintained at the Anne McLaren Building, Cambridge Biomedical Campus, under Home Office Project Licence PP4690795, *Olig2-cre*^35^ and *Sox10 fl/fl*^36^ were maintained and bred in Erlangen with 12 h dark/light cycle, water and standard chow ad libitum. Mice were genotyped using in-house PCR primers (St18-genotyping_F: GGAGCATGTTCACCTTCTGG, St18-genotyping_R: CTCCTTTCAGGTCTTGGTGA, Cre-genotyping_F: GCGGTCTGGCAGTAAAAACTATC, Cre-genotyping_R: GTGAAACAGCATTGCTGTCACTT) or using Transnetyx automated genotyping platform. *Olig2-cre Sox10 fl/fl* mice genotyping was performed as previously described in Finzsch *et al.*

### Tissue collection and cryosectioning.

Mice were euthanised with an overdose of pentobarbital. For mice older than P14, the mice were transcardially perfused with PBS followed by 4% PFA in PBS. Whole brains and spines were dissected and fixed overnight in 4% PFA at 4 °C. Spines were decalcified in 14% EDTA (Thermo Scientific, 15575020) overnight at 4 °C, followed by removal of the spinal vertebrae. All tissue samples were then transferred to sterile 30% sucrose, incubated at 4 °C for 24 – 72 hours. The tissue was embedded in OCT, stored at −80 °C and cryosectioned on glass slides as 16 μm sections on the Cryostat (Oxford Instruments). The sections were dried overnight at RT and subsequently stored at −80 °C.

### Immunohistochemistry (IHC).

Frozen slides were thawed (RT, 3 mins) and rinsed once with PBS. Antigen retrieval was performed by heating the slides to 85 °C for 10 minutes in a water bath followed by a PBS wash. The sections were blocked and permeabilized in blocking buffer (10% NDS, 0.3% Triton X-100 in PBS) for 1 hour at room temperature. Primary antibodies (Rat anti-MBP (Bio-Rad, Aa82-87), Rabbit anti-Caspr (Abcam, ab34151) were diluted in blocking buffer, applied to the tissue sections and incubated at 4 °C overnight. The slides were washed three times with blocking buffer. Secondary Alexa Fluor antibodies (Thermofisher) were added and incubated at room temperature for 1 hour protected from light. The slides were washed three times with PBS and stained with 10 μg/ml Hoechst stain in PBS for 20 minutes at room temperature, protected from light. The slides were washed twice with PBS and excess buffer was removed with a paper towel. Roughly 50 μl of ProLong Gold antifade reagent (Life Technologies, P36930) was applied to slides followed by coverslipping with a glass coverslip. The slides were stored at 4 °C until ready for confocal imaging.

### RNAscope.

smFISH was performed using the RNAscope LS Multiplex Fluorescent Assay (ACD, 322800) following established protocols^16,37^. Briefly, glass slides containing 16 μm cryosectioned tissue were thawed at room temperature for 15 min, baked at 65 °C for 45 min, and post-fixed in 4% paraformaldehyde (PFA) for 15 min. Sections were washed twice in 1X PBS and dehydrated through an ethanol series (50%, 70%, and 100% ethanol in water; 5 min each at room temperature). Slides were fitted with Leica coverplates (S21.4611, Leica) and processed on a Leica BOND RX automated staining platform. On the BOND RX, sections underwent target retrieval using ER2 (AR9640, Leica) at 95 °C for 5 min, followed by protease treatment with ACD Protease III at 42 °C for 20 min. Endogenous peroxidase activity was quenched and ACD protease III was inactivated by incubation with ACD hydrogen peroxide for 10 min at room temperature. Probe mixes were prepared by diluting C2, C3 and C4 RNAscope Z-probes in ACD C1 probe mix, namely Mm-Pdgfra-C1 (ACD, 18096A) and applied to sections for hybridization (2 h at 42 °C). The signal was amplified using sequential incubations with AMP1, AMP2, and AMP3 reagents (30 min each at 42 °C), followed by incubation with the appropriate channel-specific HRP reagent (1 min at 42 °C). TSA Opal 520, TSA Opal 570, and TSA Opal 650 (Akoya Biosciences, FP1487001KT, FP1488001KT, FP1496001KT) were applied at 1:500 dilution in TSA buffer for 30 min at 42 °C, followed by incubation with HRP blocking reagent for 15 min at 42 °C between channels. For the Atto 425 channel, sections were incubated with TSA-biotin (Akoya, NEL749A001KT; 1:500) for 30 min at room temperature, followed by streptavidin–Atto 425 (Sigma, 09260-1MG-F; 1:400) for 30 min at room temperature. Multiplex detection of up to four transcripts was achieved using probes carrying distinct channel tail configurations (C1–C4). The following probes were used in this study: Mm-Pdgfra-C1 (ACD, 18096A), Mm-Enpp6-C2 (ACD, 23335A), Mm-St18-C3 (ACD, 21201A), and Mm-Ermn-C4 (ACD, 18005A). Nuclei were counterstained with DAPI and slides were coverslipped using antifade mounting medium.

Images were acquired on an Operetta CLS High Content Analysis System (PerkinElmer) using a 40× water-immersion objective. Z-stacks were collected with a 1 μm step size and a variable number of planes. Tiled acquisitions were performed with 3% overlap and stitched using Acapella Studio version 5.3. (PerkinElmer) and then uploaded to Omero v5.6^38^ for viewing.

### RNA isolation and RT-qPCR.

Hindbrain regions were removed from freshly dissected adult mouse brains, homogenised with a sterile scalpel and resuspended in 1 ml of TRI Reagent (Zymo, R2050-1). The samples were snap frozen on dry ice and stored at −80 °C until ready for use. RNA was isolated with the RNA mini/micro kit (Zymo, R2051) according to the manufacturer’s instructions. RNA concentration was quantified using the NanoDrop^™^ 2000 (Thermo), and equal masses of RNA were reverse transcribed for cDNA synthesis using the cDNA kit (Zymo, R3012) according to the manufacturer’s instructions. For RT-qPCR, primers (St18-qPCR_F: AGCTTAAGTGTCCAACCCCG, St18-qPCR_R: GATGCCACCTTTCCTTGCAC), nuclease-free water, SYBR Green Master Mix (Thermo Fisher, A25780) and cDNA were mixed according to the manufacturer’s instructions. RT-qPCR was performed on QuantStudio 7 Flex Real-Time PCR System (Thermo). The RT-qPCR data was processed using the QuantStudio 12K flex Software and mRNA fold change values were calculated in Microsoft Excel.

### Luciferase assay.

Luciferase reporter experiments were conducted in HEK293T cells (ATCC, CRL-1573). The cell lines were cultured in high-glucose DMEM (Gibco, 11965092) supplemented with 10% fetal calf serum (FCS; Gibco, 10500064) and 1% penicillin-streptomycin (Gibco, 15140122). Transfections were performed in 24-well plates using the Xfect transfection reagent (Takara Bio, 631318). Each well received 0.5 μg of a pCMV5-based expression plasmid and 0.5 μg of a luciferase reporter construct. Where required, the total DNA mass per well was equalised by supplementing with empty pCMV5 vector. Cells were harvested 48 hours post-transfection, and whole-cell lysates were prepared in lysis buffer containing 88 mM MES (pH 7.8), 88 mM Tris (pH 7.8), 12.5 mM magnesium acetate, 2.5 mM ATP, 1 mM DTT, and 0.1% Triton X-100. Luminescence was measured using a luciferase assay buffer consisting of 0.5 mM luciferin in 5 mM KH_2_PO_4_ (pH 7.8), and chemiluminescence was detected using a luminometer.

### Statistical Analysis.

Data are presented as mean ± S.E.M. or mean ± S.D., as indicated in the figure legends, from at least three independent experiments. Sample sizes were determined based on previous studies and experimental experience. Statistical analyses were performed using GraphPad Prism. For comparisons, two-tailed unpaired Student’s t-tests or one-way/two-way ANOVA were performed, as appropriate. A P value < 0.05 was considered statistically significant.

## Figures and Tables

**Figure 1.**
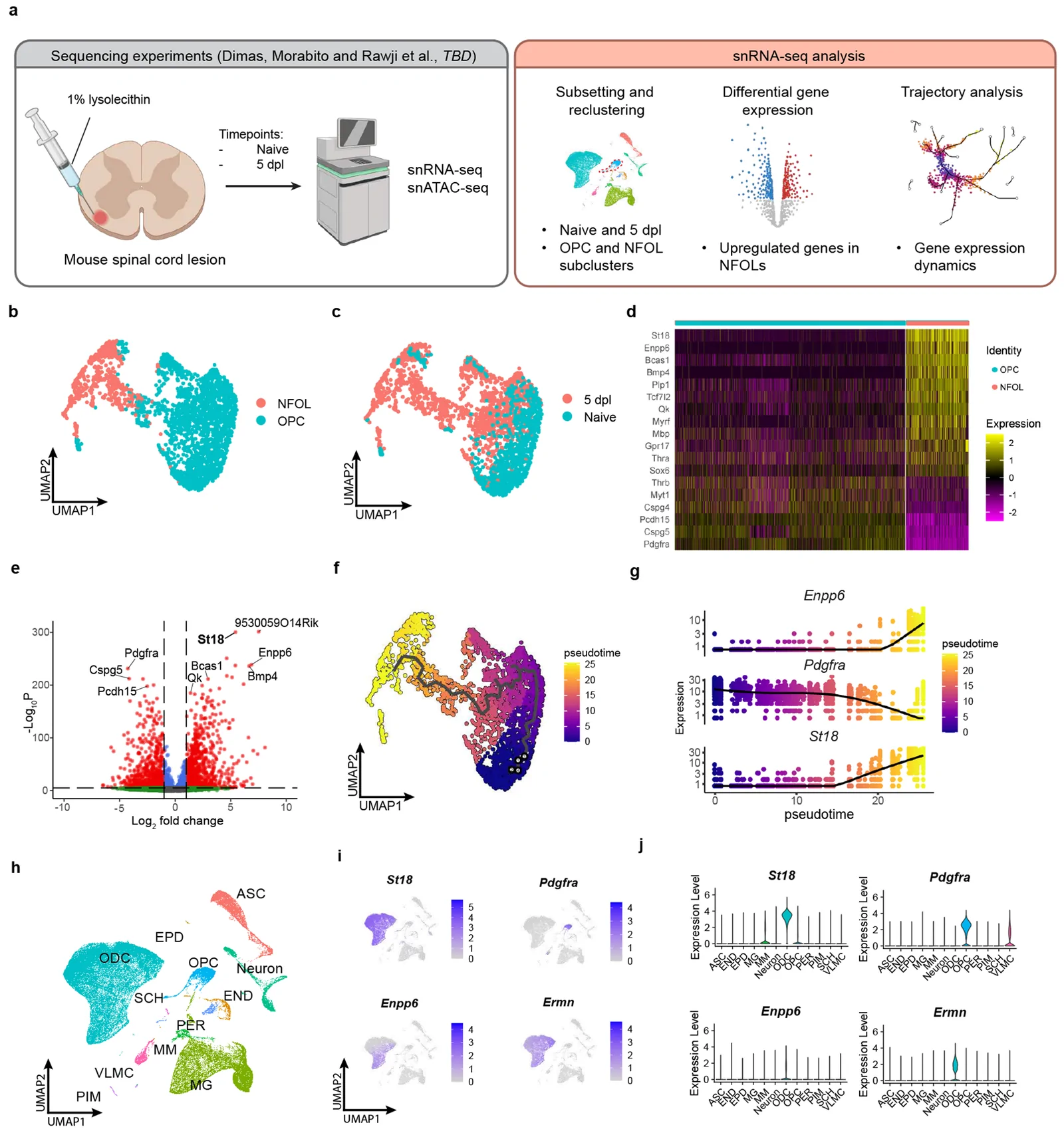
Single-nucleus transcriptomic analysis identifies ST18 induction during the OPC-to-NFOL transition. **a**, Schematic of the analytical workflow used to investigate snRNA-seq data from naïve mouse spinal cord and 5 days post-lysolecithin lesion (dpl), n=2 per group. **b-c**, UMAP projection of OPCs and NFOLs, coloured by cluster identity (b) and lesion time point (naïve vs 5 dpl) (c), showing lesion-associated expansion of NFOLs, 2,456 total nuclei; 1,931 OPC nuclei; 525 NFOL nuclei. **d**, Scaled gene expression heatmap of cell type marker genes, grouped by OPC and NFOL subclusters. **e**, Volcano plot showing differentially expressed genes between OPCs and NFOLs (red: significantly regulated in NFOLs; adjusted *p* < 0.05, log_2_FC > 1). **f**, Pseudotime trajectory analysis of the OPC-to-NFOL transition coloured by the assigned pseudotime value. **g**, Gene expression dynamics of canonical NFOL gene *Enpp6*, canonical OPC gene *Pdgfra*, and *ST18* across pseudotime. **h**, UMAP projection of all spinal cord cell types. ASC, astrocytes; END, endothelial cells; EPD, ependymal cells; MG, microglia; MM, monocyte-derived macrophage; ODC, oligodendrocytes; OPC, oligodendrocyte progenitor cells; PER, pericytes; PIM, perivascular infiltrating macrophages; SCH, Schwann cells; VLMC, vascular and leptomeningeal cells (157,563 nuclei). **i-j**, Expression of *St18*, *Pdgfra*, *Enpp6* and *Ermn* represented as UMAP projection (i) or violin plots (j).

**Figure 2.**
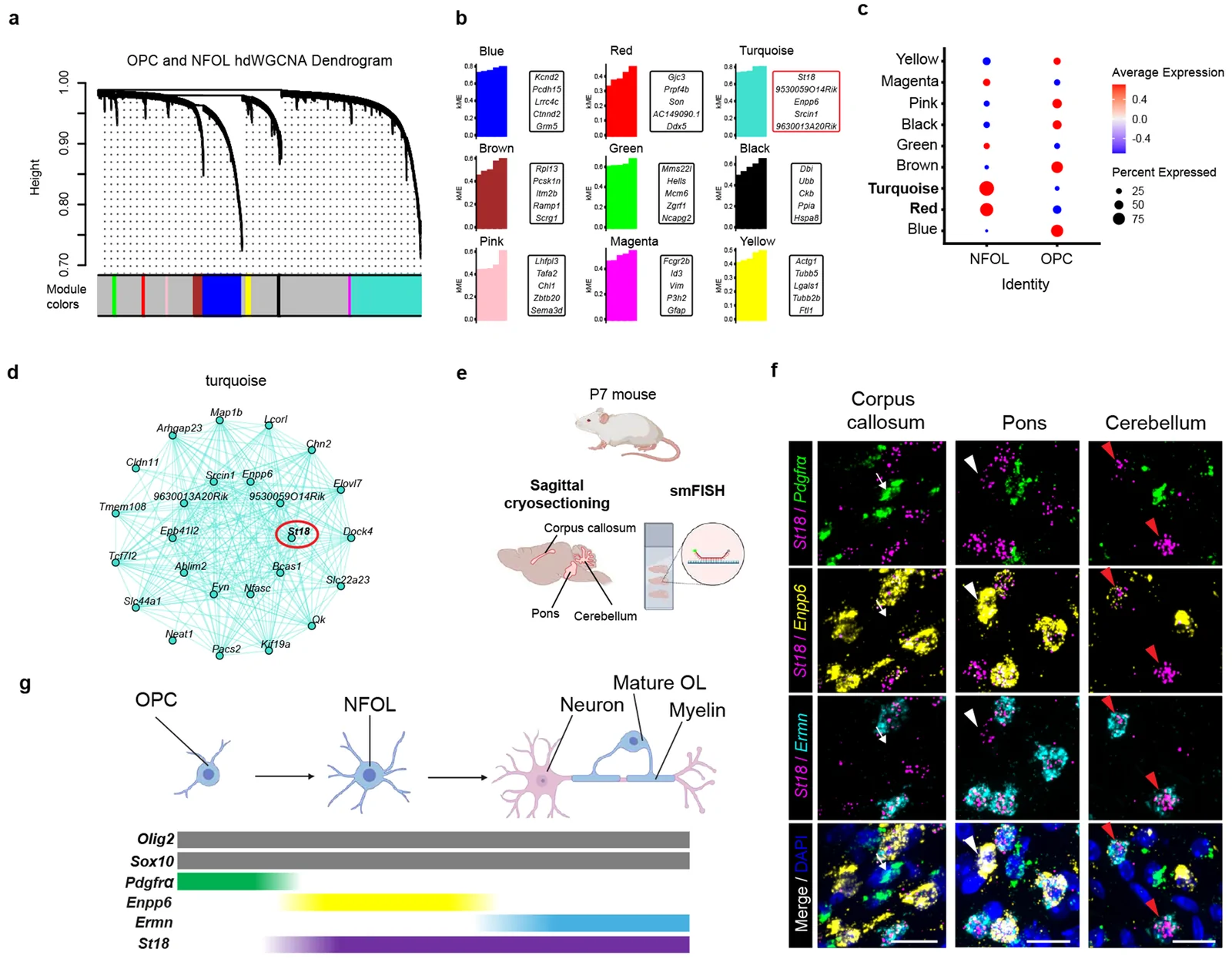
St18 defines a core transcriptional program of NFOLs. **a**, Cluster dendrogram and module detection from High-dimensional weighted gene co-expression network analysis (hdWGCNA) of OPC and NFOL subclusters. **b**, Heatmap showing module eigengene expression and the top five hub genes ranked by module membership (kME) for each hdWGCNA module. **c**, Dot plot of module eigengene (ME) enrichment across subclusters. **d**, Module-specific co-expression networks for the turquoise module. The top 10 hub genes, ranked by kME, are positioned at the centre. **e**, Single-molecule Fluorescence In Situ Hybridization (smFISH) analyses of P7 mouse brain regions (corpus callosum, Pons, Cerebellum). **f**, Representative images of spatiotemporal expression profiling using smFISH of *St18*, *Pdgfrα*, *Enpp6* and *Ermn* transcripts in the corpus callosum, pons, and cerebellum. White arrow indicates OPC (*Pdgfra*-pos. / *Enpp6*-neg. / *Ermn*-neg.), White arrowhead indicates NFOL (*Pdgfra*-neg. / *Enpp6*-pos. / *Ermn*-neg.), Red arrowhead indicates mature OL (*Pdgfra*-neg. / *Enpp6*-neg. / *Ermn*-pos.). Scale bar: 20 μm. **g**, Schematic depicting the temporal expression dynamics of key oligodendrocyte lineage markers (*Olig2*, *Sox10*, *Pdgfra*, *Enpp6*, *Ermn* and *St18*)

**Figure 3.**
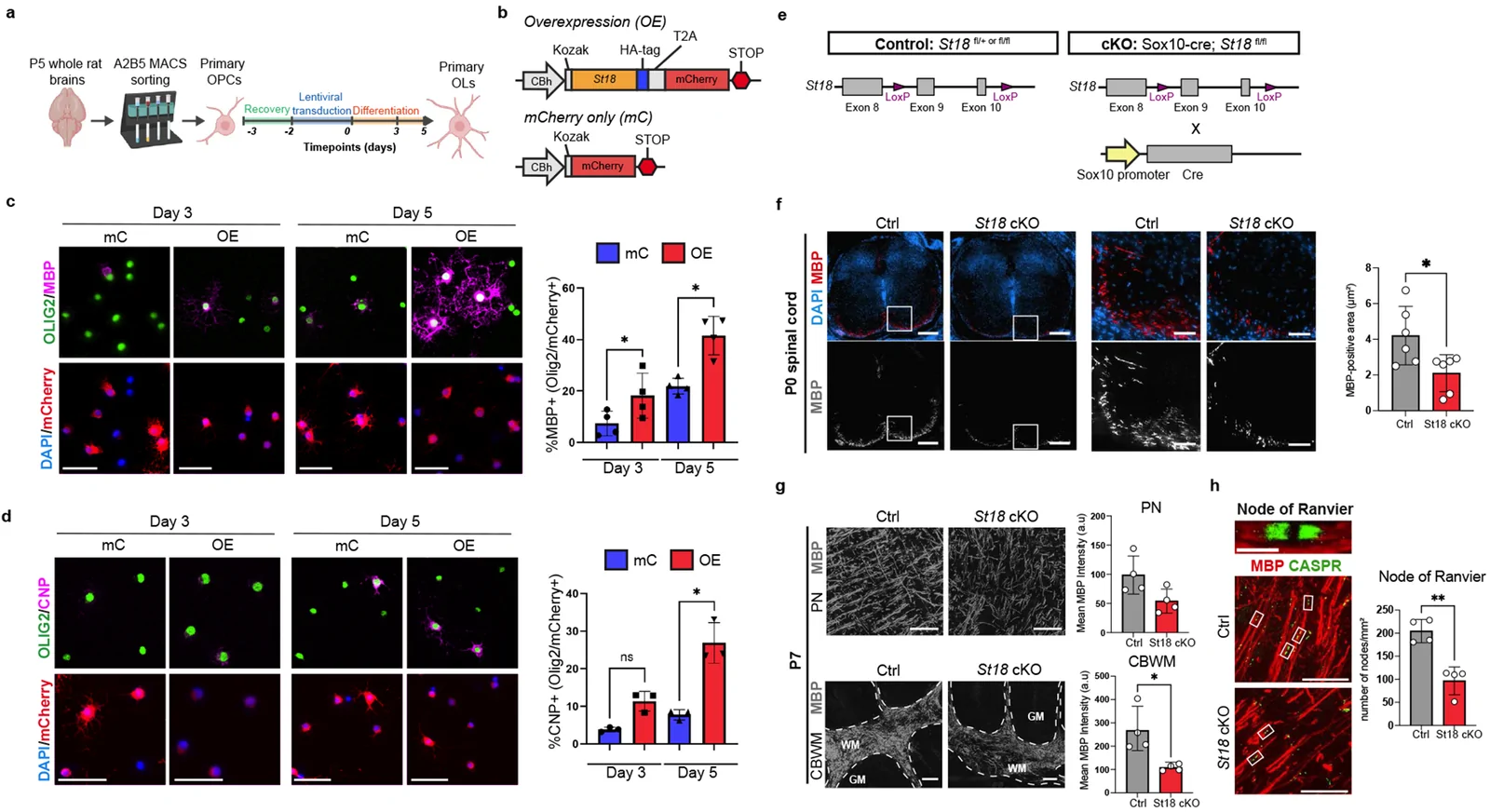
ST18 promotes oligodendrocyte differentiation and is required for timely myelin formation *in vivo*. **a**, Schematic overview of the OPC isolation, lentiviral transduction and differentiation protocol. **b**, Schematic depicting the construct to overexpress *St18* with HA tag and mCherry reporter under CBh promoter (OE) and control mCherry construct (mC). **c-d**, Immunofluorescence imaging of MBP (c) or CNP (d) together with Olig2, mCherry and DAPI of primary rat oligodendrocytes overexpressing *St18* (OE) or mCherry control (mC) with quantification (n=4 (c), n=3 (d)). **e**, Schematic representation of exon 8-10 of *St18* fl/fl mice (Control) or Sox10-cre; *St18* fl/fl mice (cKO). **f**, Immunofluorescence imaging of MBP together with DAPI of *ST18* cKO and control P0 mouse spinal cords with quantification (n=6). Right panels are zoomed from white square. **g**, MBP Immunofluorescence imaging of Pons (PN) and cerebellum white matter (CBWM) of *ST18* cKO and control P7 mice with quantification (n=4). WM, white matter; GM, grey matter. **h**, Immunofluorescence imaging of CASPR together with MBP of a node of Ranvier (top panel) and the Pons of *ST18* cKO and control P7 mice with quantification (n=4). Data are represented as mean +/− SEM. Significance was assessed using the two-tailed unpaired Student’s t-test. *, *p* < 0.05; **, *p* < 0.01; ns, not significant. Scale bar panel c, d and g: 100 μm; panel f: 250 μm and 50 μm in zoom images; panel h: 50 μm.

**Figure 4.**
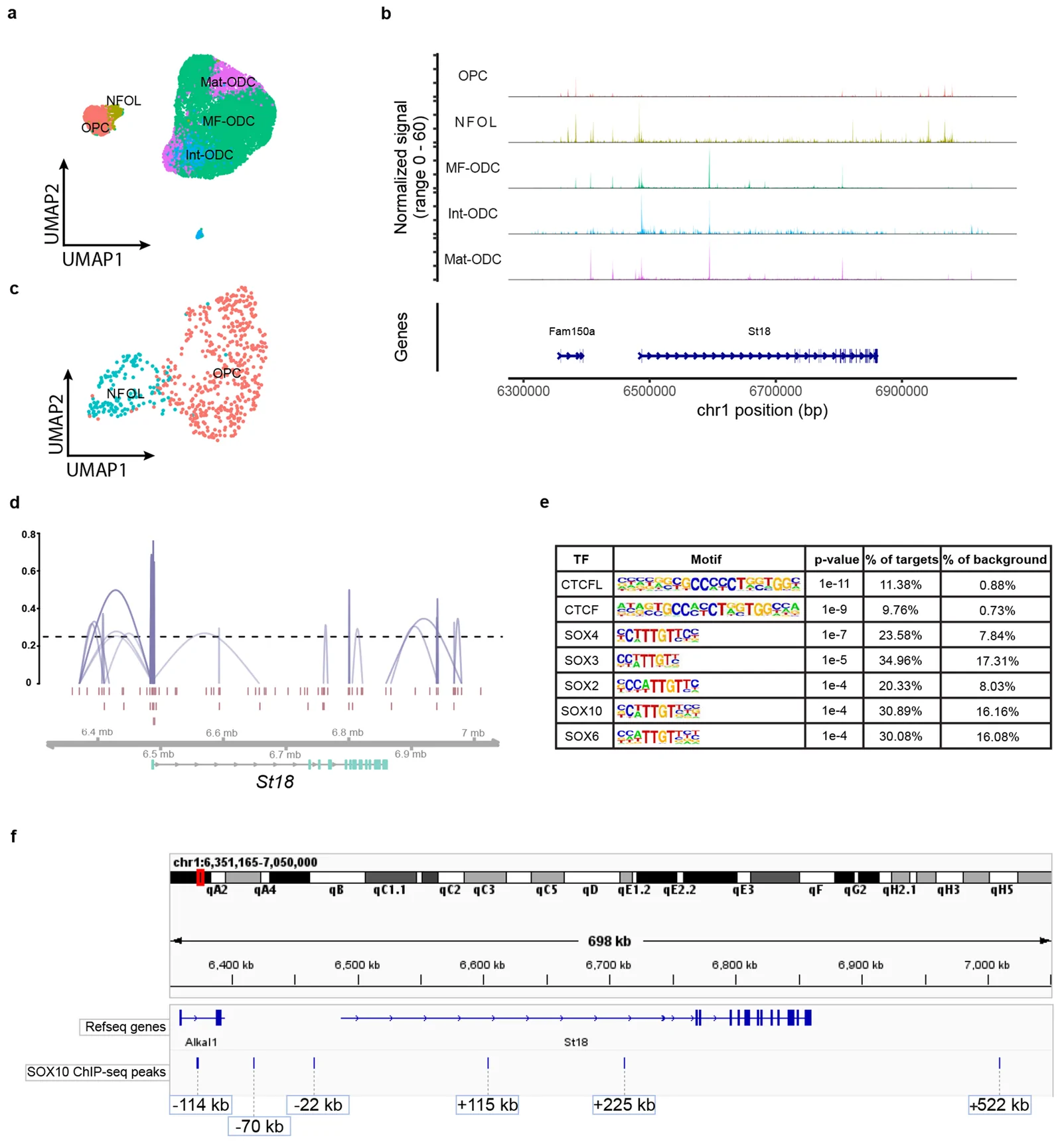
Chromatin accessibility analysis identifies putative SOX10-regulated enhancers at the St18 locus. **a**, UMAP projection of snATAC-seq profiles from all spinal cord oligodendrocyte lineage cell types. Int-ODC, intermediate oligodendrocytes; MF-ODC, myelin forming oligodendrocytes; Mat-ODC, mature oligodendrocytes. **b**, Pseudo-bulk chromatin accessibility profiles for each oligodendroglial subcluster across the St18 locus and flanking regions (chr1:6,312,485–7,045,504, mm10). Tn5 insertion signal is normalized for sequencing depth and cell number in each group. **c**, UMAP projection of OPCs and NFOLs from young naïve and 5 dpl timepoints. 608 nuclei were profiled. **d**, Co-accessible loci identified using the Cicero algorithm, comparing NFOLs to OPCs. Red bars indicate peak calls; arcs represent co-accessibility scores (threshold > 0.25, black dashed line). **e**, Motif enrichment analysis of co-accessible regions linked to the St18 transcriptional start site (*TSS*). **f**, ChIP-seq profiles from P15 rat spinal cord homogenates showing SOX10 binding near the St18 locus. Peaks are based on data from Lopez-Anido *et al.* (2015) (GEO ID: GSE64703), with six putative regulatory regions highlighted relative to the St18 transcription start site (TSS): −114 kb, −70 kb, −22 kb, +115 kb, +225 kb, and +522 kb. Genomic coordinates are chr1:6351165-7050000.

**Figure 5.**
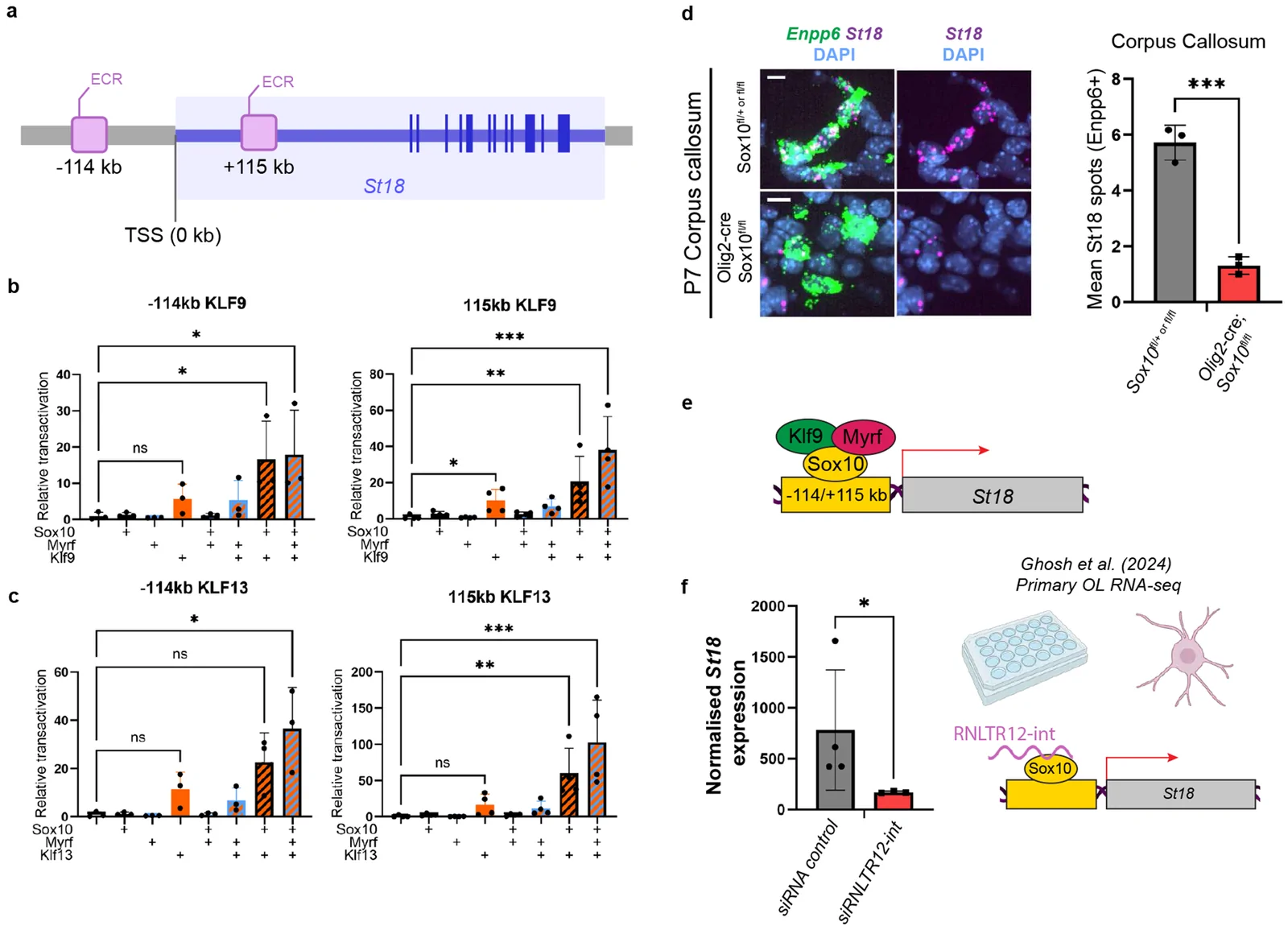
SOX10, MYRF and KLF9/13 cooperatively regulate St18 transcription through distal cis-regulatory elements. **a**, Schematic overview of the *St18* locus showing the relative genomic positions of SOX10 binding sites. Purple boxes indicate the SOX10 binding sites that contain evolutionarily conserved regions (ECR) or putative cis-regulatory regions (CRE). **b-c**, Luciferase reporter assays showing the transcriptional activity of the putative SOX10-bound regulatory regions located at −114 kb (left) and +115 kb (right) relative to the *St18* TSS following expression of SOX10 in combination with MYRF and KLF9 (b) or KLF13 (c). Luciferase activity is presented relative to the control condition and normalized to the empty vector (n=3,4). **d**, smFISH for St18 and Enpp6 mRNA in the corpus callosum of P7 Sox10 cKO (Olig2-cre;Sox10fl/fl) and control mice (Sox10fl/+ or fl/fl), with quantification (n=3). **e**, Schematic model of transactivation of *St18* by SOX10 in cooperation with MYRF and KLF9/13 through upstream and intronic enhancer elements (−114 kb and +115 kb). **f**, Relative *St18* mRNA expression following RetroMyelin (RNLTR12-int) knockdown in differentiating OPCs, analysed from published bulk RNA-seq data^17^ (n=3,4).

**Figure 6.**
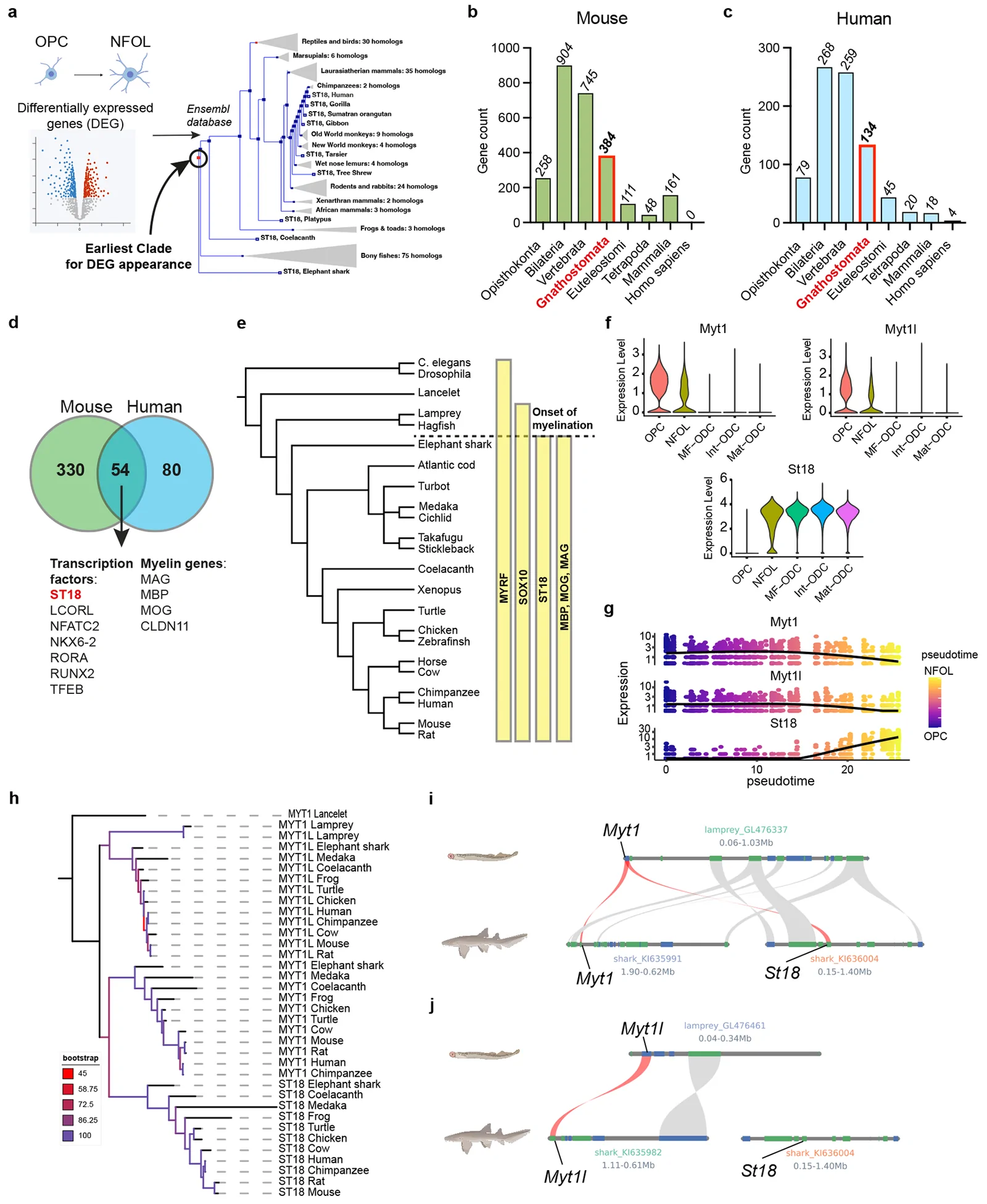
Evolutionary analyses link the emergence of ST18 to the origin of myelin in jawed vertebrates. **a**, Schematic overview of the comparative genomics workflow used to identify the evolutionary origin of genes differentially expressed during the OPC-to-NFOL transition. **b-c**, Phylostratigraphic analysis of differentially expressed genes according to their earliest evolutionary appearance across major metazoan lineages in mouse (b) and human (c). Bars represent the number of genes originating within each clade. The emergence of jawed vertebrates (Gnathostomata), corresponding to the evolutionary appearance of myelin, is highlighted in red. **d**, Venn diagram showing conserved genes shared between mouse and human that first emerged in Gnathostomata and are differentially expressed during the OPC-to-NFOL transition. Conserved genes shared between species (n = 54) are indicated, including transcription factors and myelin-associated genes. **e**, Evolutionary timeline showing the emergence of oligodendrocyte regulatory and myelin-associated genes across major metazoan lineages. The onset of myelination is indicated by the dashed line. **f**, Violin plots showing expression of *Myt1*, *Myt1l* and *St18* across oligodendrocyte lineage from mouse spinal cord snRNA-seq data. OPC, oligodendrocyte precursor cells; NFOL, newly formed oligodendrocytes; MF-ODC, myelin-forming oligodendrocytes; Int-ODC, intermediate oligodendrocytes; Mat-ODC, mature oligodendrocytes. **g**, Pseudotime expression dynamics of *Myt1, Myt1l* and *St18* during the OPC-to-NFOL transition. **h**, Maximum-likelihood phylogenetic tree of vertebrate MYT family proteins showing the evolutionary relationships between Myt1, Myt1l, and St18 orthologues. Branch lengths are proportional to amino acid substitutions per site, and branch colours indicate bootstrap support values. **i-j**, Microsynteny analysis of *Myt1*, *St18*, and *Myt1l* loci across lamprey and elephant shark. i, Conserved synteny between ancestral Myt1 loci in lamprey and corresponding Myt1 and St18 loci in elephant shark. j, Comparison of Myt1l loci between lamprey and elephant shark showing conserved synteny restricted to Myt1l. Grey links indicate collinear gene pairs, whereas red links highlight conserved syntenic relationships between Myt1 and St18 loci.

## Data Availability

The data that support the findings of this study are available from the corresponding author upon request. Custom analysis code for this study is available on GitHub (https://github.com/danyama312/St18_oligodendrocytes).
